# An inductive graph neural network model for compound–protein interaction prediction based on a homogeneous graph

**DOI:** 10.1093/bib/bbac073

**Published:** 2022-03-12

**Authors:** Xiaozhe Wan, Xiaolong Wu, Dingyan Wang, Xiaoqin Tan, Xiaohong Liu, Zunyun Fu, Hualiang Jiang, Mingyue Zheng, Xutong Li

**Affiliations:** State Key Laboratory of Drug Research, Drug Discovery and Design Center, Shanghai Institute of Materia Medica, Chinese Academy of Sciences, 555 Zuchongzhi Road, Shanghai 201203, China; University of Chinese Academy of Sciences, No.19A Yuquan Road, Beijing 100049, China; State Key Laboratory of Drug Research, Drug Discovery and Design Center, Shanghai Institute of Materia Medica, Chinese Academy of Sciences, 555 Zuchongzhi Road, Shanghai 201203, China; School of Pharmacy, East China University of Science and Technology, Shanghai 200237, China; State Key Laboratory of Drug Research, Drug Discovery and Design Center, Shanghai Institute of Materia Medica, Chinese Academy of Sciences, 555 Zuchongzhi Road, Shanghai 201203, China; University of Chinese Academy of Sciences, No.19A Yuquan Road, Beijing 100049, China; ByteDance AI Lab, Shanghai 201103, China; AlphaMa Inc., No. 108, Yuxin Road, Suzhou Industrial Park, Suzhou 215128, China; State Key Laboratory of Drug Research, Drug Discovery and Design Center, Shanghai Institute of Materia Medica, Chinese Academy of Sciences, 555 Zuchongzhi Road, Shanghai 201203, China; State Key Laboratory of Drug Research, Drug Discovery and Design Center, Shanghai Institute of Materia Medica, Chinese Academy of Sciences, 555 Zuchongzhi Road, Shanghai 201203, China; University of Chinese Academy of Sciences, No. 19A Yuquan Road, Beijing 100049, China; School of Life Science and Technology, ShanghaiTech University, 393 Huaxiazhong Road, Shanghai 200031, China; State Key Laboratory of Drug Research, Drug Discovery and Design Center, Shanghai Institute of Materia Medica, Chinese Academy of Sciences, 555 Zuchongzhi Road, Shanghai 201203, China; University of Chinese Academy of Sciences, No. 19A Yuquan Road, Beijing 100049, China; State Key Laboratory of Drug Research, Drug Discovery and Design Center, Shanghai Institute of Materia Medica, Chinese Academy of Sciences, 555 Zuchongzhi Road, Shanghai 201203, China; University of Chinese Academy of Sciences, No. 19A Yuquan Road, Beijing 100049, China

**Keywords:** compound–protein interaction prediction, homogeneous graph, end-to-end learning, inductive graph neural network

## Abstract

Identifying the potential compound–protein interactions (CPIs) plays an essential role in drug development. The computational approaches for CPI prediction can reduce time and costs of experimental methods and have benefited from the continuously improved graph representation learning. However, most of the network-based methods use heterogeneous graphs, which is challenging due to their complex structures and heterogeneous attributes. Therefore, in this work, we transformed the compound–protein heterogeneous graph to a homogeneous graph by integrating the ligand-based protein representations and overall similarity associations. We then proposed an Inductive Graph AggrEgator-based framework, named CPI-IGAE, for CPI prediction. CPI-IGAE learns the low-dimensional representations of compounds and proteins from the homogeneous graph in an end-to-end manner. The results show that CPI-IGAE performs better than some state-of-the-art methods. Further ablation study and visualization of embeddings reveal the advantages of the model architecture and its role in feature extraction, and some of the top ranked CPIs by CPI-IGAE have been validated by a review of recent literature. The data and source codes are available at https://github.com/wanxiaozhe/CPI-IGAE.

## Introduction

Identification of potential compound–protein interactions (CPIs) plays an essential role in drug hit identification, understanding drug side effects, and finding new indications of existing drugs [1, 2]. However, it is also a costly, laborious and time-consuming step through wet-lab experiments due to the need of searching over large compound space [3]. Computational approaches can significantly reduce the time and costs of experimental methods, and thus, it is of high interest to develop computational models that can provide reliable CPI candidates for the biologists. Among the traditional *in silico* methods for predicting CPIs, one commonly used is molecular docking [4–6]. Although remarkable improvements have been made in this area, practical challenges are still open, such as protein structural flexibility, appropriate scoring function and high requirement of computational resources [3].

In the past decade, with massive biomedical data being collected and accessible, along with the advances of data mining technologies, which have been successfully applied in many areas, numerous data-driven computational methods have been developed rapidly for CPI prediction [2, 3, 7]. According to the types of input data, these methods can be roughly divided into feature-based methods and network-based methods [8].

Feature-based methods feed descriptors that represent the features of compounds and proteins to the downstream machine learning algorithms to model CPI. Commonly used descriptors for compounds are molecular fingerprints that include the extended connectivity fingerprints (ECFPs) [9], the Molecular ACCess System (MACCS) keys [10] and so on. For proteins, the available descriptors include the composition–transition–distribution descriptors [11], the position-specific scoring matrix (PSSM) [12] and so on. With the wide application of natural language processing methods, sequential features such as the simplified molecular input line entry system (SMILES) [13] for compounds and the amino acid sequences for proteins can be directly used as input to the downstream models. Different statistical methods were applied as the data-mining algorithm at the early stage. The similarity ensemble approach (SEA) [14] relates proteins based on the statistically calculated similarity of their respective ligands, which was further applied for target identification successfully [15]. Many machine learning models were also proposed to mine the similarity from input features, with improved predictive performance. For instance, TarPred [16, 17] integrates the *k*-nearest neighbors (KNN) algorithm with the molecular similarity-based searching strategy for target identification and shows a significant improvement compared with the SEA. Recently, deep learning algorithms have shown further enhancement due to their capability to explore complex nonlinear information behind the input features. Various effective deep learning-based models were proposed to predict potential CPIs, such as deep belief networks in DeepDTIs [18], convolutional neural networks in DeepDTA [19], tranformer architecture in TransformerCPI [2] and so on. These feature-based methods improve the accuracy of CPI prediction to some extent and can be generalized to the CPIs outside their training dataset due to their relatively strong scalability. However, these methods do not take compound–compound similarities and protein–protein interactions into account explicitly [20].

Network-based methods first construct a network from the collected dataset and then use the graph-related algorithms to explore useful information from the network for CPI prediction. The network can describe the interactions between various biological entities, such as compounds and proteins. Bipartite graphs are a frequently used structure [21], and more complex heterogeneous graphs including more relation types (e.g., drug–disease relations and target–disease relations) have been proposed [22–25]. Although a heterogeneous graph can integrate multiple types of entities and interactions in a single network, it is still challenging to aggregate heterogeneous attributes of different types of nodes or edges to obtain the graph representation [26]. In the early stage, network propagation algorithms were used for feature extraction, such as random walk with restart in DTINet [22]. The extracted features are then used as inputs of simple machine learning models to predict CPIs, such as the Hybrid algorithm in DT-Hybrid [27], the support vector machine (SVM) in Bipartite Local Model with Neighbor-based Interaction-profile Inferring (BLMNII) [21] and the matrix completion in DTINet [22]. These models show moderate performance partly because of their lack of the nonlinear expressive power. Furthermore, the feature extraction and the CPI prediction of these models are independent steps, i.e. the parameters involved in the network propagation algorithms cannot be optimized by the CPI prediction task [26]. In recent years, graph neural networks (GNNs) have been utilized in extracting representations for heterogeneous graphs, such as graph convolutional networks in NeoDTI [23], and graph convolutional autoencoders and generative adversarial networks (GANs) in GANDTI [28]. Deep models have shown stronger performance than these two-step methods in CPI prediction. However, as GNNs were designed to process the homogeneous graph, they project the nodes of different types into a common feature space via direct aggregation and concatenation, which may lead to substantial loss of the valuable heterogeneous information. Moreover, most of these graph-related algorithms are transductive and cannot be easily adapted to CPIs outside the dataset.

To overcome the drawbacks of heterogeneous graphs, we transformed the compound–protein heterogeneous graph to a homogeneous graph with directed and weighted edges by integrating the ligand-based protein representations and overall similarity associations. The ligand-based representation has been proved to be an efficient way for protein characterization [29] and has been widely used such as in TarPred and SEA. The specially designed homogeneous graph can simplify the graph structure and maintain the consistency of node and edge attributes, which is beneficial for message aggregating and updating in the graph, as most GNN operations are aimed at homogeneous graphs. In addition, the IGAEs for representation learning, which were adapted from the inductive GNN GraphSAGE, makes it possible to predict CPIs outside the dataset. Thus, we propose a novel inductive GNN model, named CPI-IGAE, to identify CPIs based on a specially designed homogeneous graph. Via comprehensive comparisons of performance, we show that our model outperforms some feature-based methods and network-based methods and has competitive performance with molecular docking. The ablation study and visualization of embeddings further demonstrate the effectiveness of our method. Moreover, some of the top ranked CPIs have been validated by a review of recent literature, which indicates its ability to provide potential CPI candidates for further explorations.

In summary, the major contributions of this research are summarized as follows:

(i) To better conduct message passing and aggregating in graph, we transformed the heterogeneous graph to a homogeneous graph with directed and weighted edges.
(ii) We adapted the inductive aggregators from GraphSAGE to fit the CPI prediction task and this enables our methods to predict CPIs outside the modeling dataset, which improves the generalization ability of this method.
(iii) We proposed an end-to-end framework, which can help learn the task-specific node embeddings for CPI prediction.
(iv) The comprehensive performance evaluations of this model indicate that CPI-IGAE outperforms some state-of-the-art CPI prediction methods.

## Methodology

**Figure 1:**
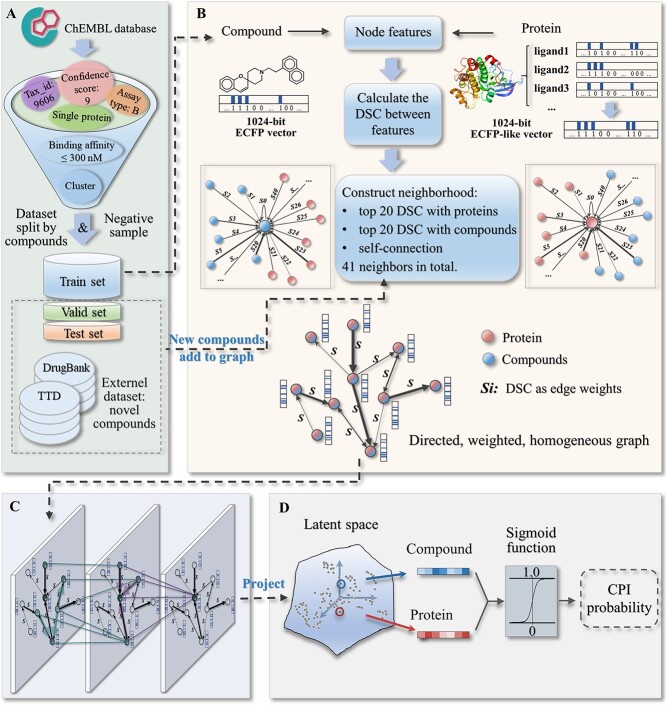
The workflow of CPI-IGAE. (**A**) Collection of the modeling dataset. (**B**) Construction of a homogeneous graph with directed and weighted edges. (**C**) IGAEs to project the nodes into a latent space. (**D**) Discriminator to transform the node embeddings in the latent space to probabilities of CPI.

As shown in Figure 1, this work can be divided into four parts: (i) collection of modeling dataset; (ii) construction of a homogeneous graph with directed and weighted edges; (iii) IGAEs for obtaining the low-dimensional node embeddings and (iv) a discriminator for CPI identification.

### Collection of modeling dataset

As shown in Figure 1A, the modeling dataset was collected from ChEMBL (v23). Here, only CPIs meeting the following criteria were used:

(i) the protein tax_id is 9606, which means the sequence is derived from human;
(ii) the protein type is ‘single protein’;
(iii) the assay relationship type is ‘B’, which means a direct protein–ligand interaction;
(iv) the target confidence score is 9, which indicates a direct assignment of single protein target to the ChEMBL ligand with a high degree of confidence and
(v) the bioactivity type is *K*_i_, *K*_d_, IC_50_ or EC_50_, and the activity value is better than 300 nM using relation ‘=’ or ‘<’.

Then, data cleaning was made to merge the compounds which have same structures but different ChEMBL ids, and the same operation was used for the protein targets. Next, the compounds were clustered according to their ECFPs with a distance cutoff of 0.4 using RDKit [30], and the center point of each compound cluster was selected. The purpose of this operation is to avoid the ‘analogue bias’ [31], which means that the predictive ability can be improved artificially if the modeling dataset contains many compounds with the same chemotypes. Subsequently, to ensure that the protein targets within the training set can be efficiently represented by their ligands, the proteins with less than five ligands were removed. Finally, a total of 55 212 CPIs including 34 908 compounds and 784 protein targets were used to train the model.

The 34 908 compounds were randomly split into training, validation and test set with a ratio of 8:1:1, with guaranteeing that each protein has at least one ligand in each set. Finally, the training set has 29 058 compounds with 42 025 CPIs, the validation set has 2939 compounds with 6566 CPIs and the test set has 2911 compounds with 6621 CPIs.

### Construction of a homogeneous graph

As shown in Figure 1B, a homogeneous graph with directed and weighted edges was designed to organize the collected dataset.

In the training process, 42 025 CPIs involving 29 058 compounds and 784 proteins in the training set were used to construct a graph of 29 842 nodes to learn the parameters of CPI-IGAE. (i) For compound nodes, the feature is the 1024 bit ECFPs. (ii) For protein nodes, the 1024 bit ECFP-like vector is constructed by its ligands in the training set. More specifically, for each bit of a protein feature vector, if more than one-third of the bit of its ligands in the training set is 1, it is set to 1, otherwise 0. The value ‘1/3’ was chosen due to its best performance (Supplementary Document Section 1.1 available online at http://bib.oxfordjournals.org/).

Edges in this graph were constructed by the Dice similarity coefficients (DSCs) [32] between the node features. For each node, 40 incoming edges to it were constructed from the top 20 compound nodes and the top 20 protein nodes with DSCs to it, because choosing top 20 has been tested to show the best tradeoff between model performance and runtime (Supplementary Document Section 1.2 available online at http://bib.oxfordjournals.org/). The corresponding DSC values were used as weights of these directed edges. Besides, a self-connected edge was set for each node with a weight of 1. Until now, we have constructed a homogeneous graph with directed and weighted edges.

In the testing process, the DSCs of the new compounds relative to all nodes in the modeling graph need to be calculated. Then, the top 20 compound nodes and protein nodes with the largest DSCs will be chosen to construct the edges pointing to the new compounds, and thus, the new compounds can be added to the modeling graph to form a larger inference graph for testing.

### IGAE**s for feature extraction**

The IGAE inspired by GraphSAGE enables the CPI-IGAE to predict new CPIs outside the modeling dataset. Some adjustments were made for the GraphSAGE aggregators to better meet our requirements (see Supplementary Document Section 2 available online at http://bib.oxfordjournals.org/). For each node $v$ in the k-th aggregator, all the previous embeddings ${h}_{u_i}^{k-1}$ from its full neighbor set $N(v)$ are first aggregated together through a max-pooling operation according to:

(1) \begin{equation*} {h}_{N(v)}^k=\max \left(\left\{\sigma \left({W}_{\mathrm{pool}}\bullet{s}_{u_iv}\bullet{h}_{u_i}^{k-1}+b\right),\forall{u}_i\in N(v)\right\}\right), \end{equation*}

where the weight ${s}_{u_iv}$ of the directed edge from node ${u}_i$ to $v$ is multiplied by the learnable pooling matrices ${W}_{\mathrm{pool}}$ for neighborhood information aggregation. Edge weights can provide useful initial information to improve the accuracy and speed of training, and the directed edges can prevent information redundancy caused by repeated aggregations. $\sigma$ is a nonlinear activation function using the rectified linear unit. Then, the aggregating information ${h}_{N(v)}^k$ is concatenated to the previous embedding ${h}_v^{k-1}$ according to

(2) \begin{equation*} {h}_v^k=\sigma \left({W}^k\bullet concat\left({h}_v^{k-1},{h}_{N(v)}^k\right)\right), \end{equation*}

where ${W}^k$ represents learnable updating matrices and ${h}_v^k$ is the updated node embedding.

Since the attributes of nodes or edges in this homogeneous graph have the same meaning, they can be fused directly through the operations. Each aggregator is in charge of one-hop distance neighbors.

### Discriminator for CPI identification

Through the IGAEs, every node in the graph is represented as a 500 bit vector, which can be regarded as being projected to a low-dimensional latent space. These information-intensive embeddings contain the structural information and the overall relationships. For the CPI prediction task, we aim to shape this latent space to follow the constraint that a compound node and a protein node would be closer to each other if there is an interaction between them. A discriminator was designed to covert the distance between a compound node and a protein node in the latent space into a probability of CPI. Given a compound–protein pair, the discriminator is defined as:

(3) \begin{equation*} \hat{y}=\sigma \left( em{b}_m\bullet em{b}_t\right). \end{equation*}

As shown above, the probability $\hat{y}$ of existing an interaction between this pair is obtained by using the sigmoid function $\sigma$ to regularize the dot product of the compound node embedding $em{b}_m$ and the protein node embedding $em{b}_t$ to a range of 0 to 1.

CPI prediction is a sparsely labeled problem, i.e. the number of negatives is much higher than that of positives, whereas positive samples greatly dominate negative samples in the most of open-sourced database. Therefore, the number of negative samples in this work was set to 10 times of the positives in order to mimic this typical application scenario [23]. An adjusted negative sampling was used to generate negative samples (Supplementary Document Section 1.3 available online at http://bib.oxfordjournals.org/). We use Focal Loss to fit the predict score and the label value [33], which is a loss function for dealing with the hard-classified examples in dense object detection, and can be regarded as a dynamic scaled cross entropy loss as below:

(4) \begin{equation*} FL=\left\{{\displaystyle \begin{array}{@{}cc}-\alpha{\left(1-p\right)}^{\gamma}\log (p),& if\ y=1\\{}-\left(1-\alpha \right){p}^{\gamma}\log \left(1-p\right), &if\ y=0\end{array}}\right. \end{equation*}

where $y$ is the true label, $p$ is the predicted result, $\gamma\ (\gamma \ge 0)$ is a tunable parameter and ${(1-p)}^{\gamma }$ is a modulating factor which can focus training on hard-classified samples. The model was optimized through grid hyperparameter searching along with an early stopping strategy. The implement details are shown in the Supplementary Document available online at http://bib.oxfordjournals.org/.

**Figure 2:**
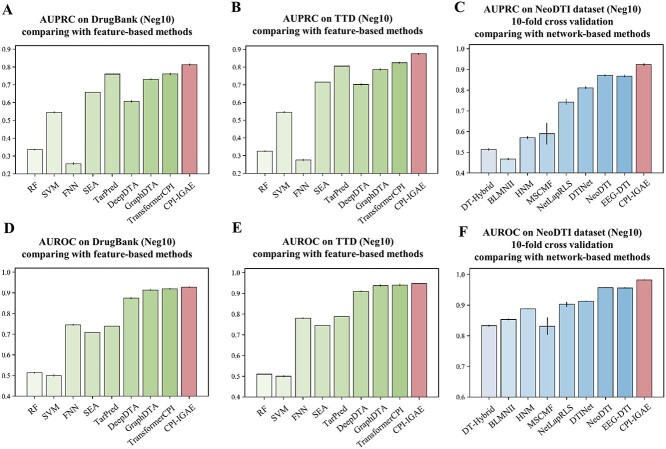
Comparison results with other methods. (**A**) Comparison with other feature-based methods on DrugBank in terms of AUPRC. (**B**) Comparison with other feature-based methods on TTD in terms of AUPRC. (**C**) Comparison with other network-based methods on the NeoDTI dataset using 10-fold cross-validation in terms of AUPRC. (**D**) Comparison with other feature-based methods on DrugBank in terms of AUROC. (**E**) Comparison with other feature-based methods on TTD in terms of AUROC. (**F**) Comparison with other network-based methods on the NeoDTI dataset using 10-fold cross-validation in terms of AUROC.

**Figure 3:**
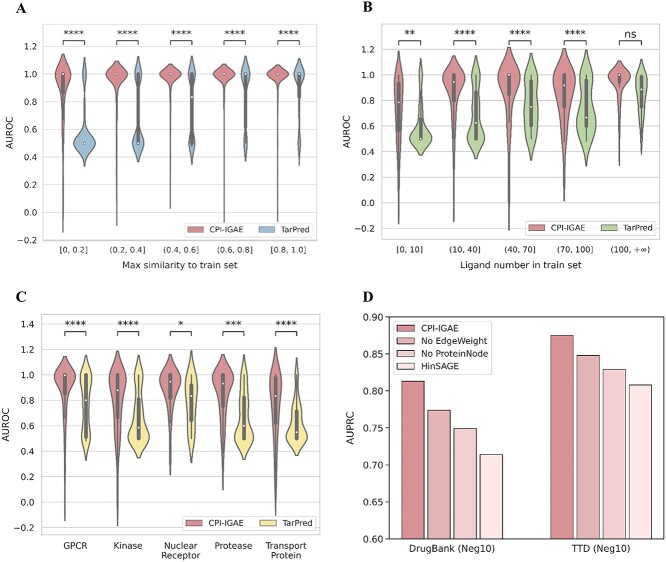
More detailed performance evaluations of CPI-IGAE. (**A**) Violine plot to show the distribution of AUROCs of CPI-IGAE (red) and TarPred (blue) for test compounds in the DrugBank dataset with the increase of their maximum Tanimoto similarities relative to the training compounds. (**B**) Violine plot to show the distribution of AUROCs of CPI-IGAE (red) and TarPred (green) for targets in the DrugBank dataset with the increase of their ligand numbers in the train set. (**C**) Violine plot to show the distribution of AUROCs of CPI-IGAE (red) and TarPred (yellow) for targets in the DrugBank dataset of different protein families. (**D**) Results of the ablation studies in terms of AUPRC in the external test set DrugBank and TTD. Note: The effects of the two models were compared using the Mann–Whitney–Wilcoxon one-tailed hypothesis test with Bonferroni correction of *P*-values ^*^^*^^*^^*^, *P* < 0.0001; ^*^^*^^*^, 0.0001 < *P* ≤ 0.001; ^*^^*^, 0.001 < *P* ≤ 0.01; ^*^, 0.01 < *P* ≤ 0.05 and ns, 0.05 < *P* ≤ 1.0.

## Results

### Model performance

CPI prediction can be treated as a binary classification task. Thus, the performance of this model was evaluated by the area under the precision-recall curve (AUPRC) and the area under the receiver-operating-characteristic curve (AUROC). The best performance model shows an AUPRC of 0.956 and an AUROC of 0.985 on the test set, which was chosen for further evaluations.

#### Comparison with feature-based methods

To evaluate the inductive generalizability of CPI-IGAE, we tested our model on two external datasets from DrugBank (v5.1.1) [34] and Therapeutic Target Database (TTD) (v6.1.01) [35], which have different distributions from the modeling dataset (Supplementary Figure S5A available online at http://bib.oxfordjournals.org/). On these two convincing external datasets, we compared CPI-IGAE with feature-based methods which can be divided into ligand-based methods and chemogenomics-based methods [36]. The formers are SEA [14, 15] and TarPred [16, 17], and the latters are self-build SVM, random forest and a fully connected neural network, as well as previous reported models including DeepDTA [19], GraphDTA [37] and TransformerCPI [2]. Details for this experiment can be found in Supplementary Document Section 4.1 and Table S2 available online at http://bib.oxfordjournals.org/. The results show that CPI-IGAE outperforms other methods according to AUROC and AUPRC (Figure 2A–B and D–E). Particularly, although the number of negative samples is 10 times of the positive ones, the AUPRC of CPI-IGAE is over 5% higher than the AUPRCs of three state-of-the-art chemogenomics-based methods, i.e. DeepDTA, GraphDTA and TransformerCPI, and 7% higher than that of the best ligand-based method TarPred, which demonstrates the convincing predictive ability of CPI-IGAE, as AUPRC can provide a more informative criterion than AUROC on imbalanced datasets [38]. Moreover, we conducted more detailed evaluations of CPI-IGAE compared with the best ligand-based method TarPred on the external DrugBank dataset.

(i) We first compared the model performance for compounds with various degrees of similarity to the training compounds, because CPI-IGAE and TarPred both use similarity information. As Figure 3A shows, the performance of TarPred becomes worse for the test compounds with low similarities to the training set while CPI-IGAE is robust to compounds with both high and low similarities to the training set. Moreover, CPI-IGAE significantly outperforms TarPred within every similarity interval.
(ii) We then compared the model performance for proteins with various number of ligands, as CPI-IGAE and TarPred both use ligand-based representation of proteins. Figure 3B shows that CPI-IGAE is robust to proteins with various number of ligands, while TarPred is biased towards the targets with more ligands. CPI-IGAE significantly outperforms TarPred with AUROCs greater than 0.8 when ligand numbers are less than 100 (Figure 3B). Because most of the ligand numbers of the target are less than 100 (Supplementary Figure S5B available online at http://bib.oxfordjournals.org/), this further confirms the applicability of CPI-IGAE.
(iii) We further compared the model performance for various protein families, because it is important to check whether the prediction model is biased towards one particular protein family. The proteins were mapped to different protein families according to the annotations in the ChEMBL (v23), and there are mainly five classes: 156 G-protein-coupled receptors (GPCRs), 180 kinases, 77 proteases, 28 nuclear receptors and 63 transport proteins (including 38 ion channels and 25 transporters). Figure 3C reveals that CPI-IGAE is robust across all of the five protein families and performs better than TarPred.

**Figure 4:**
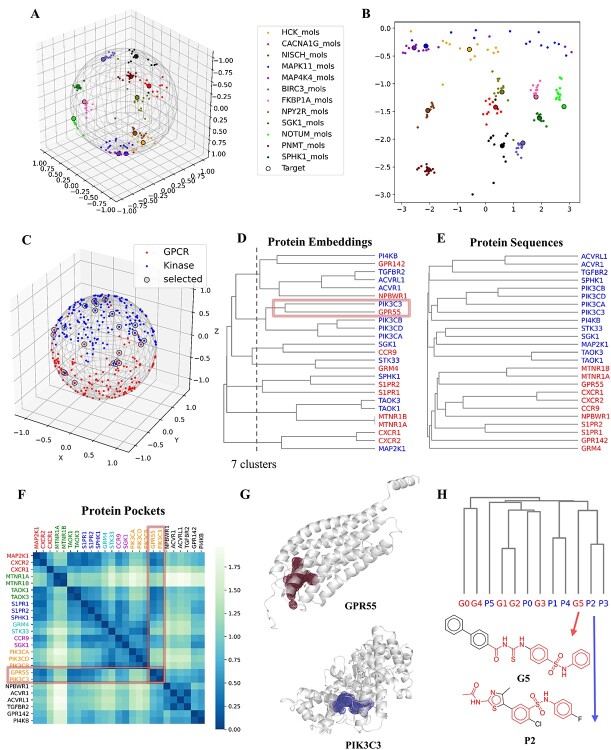
Visualization of embeddings. (**A**) Visualization of the embeddings of 12 randomly selected targets and their ligands on the surface of a hypersphere by Uniform Manifold Approximation and Projection (UMAP) with the Haversine metric. (**B**) 2D map projection of (**A**). (**C**) Visualization of the embeddings of GPCRs (red) and kinases (blue) on the surface of a hypersphere by UMAP with the Haversine metric. (**D**) The cluster tree generated from hierarchical clustering using the embeddings of 25 proteins which were randomly selected from the mixed up GPCRs and kinases in (**C**). (**E**) The phylogenetic tree generated from MSA of Clustal Omega using the amino acid sequences of the 25 proteins. (**F**) The heat map to show the Euclidean distance between the pocket vectors of the proteins, and different colors of the protein names corresponds to different clusters in (**D**). (**G**) The pockets of GPR55 (red) and PIK3C3 (blue) found by SiteMap. (**H**) The cluster tree of the ligands of G-protein coupled receptor 55 (GPR55) (red) and Phosphatidylinositol 3-kinase catalytic subunit type 3 (PIK3C3) (blue) by hierarchical clustering using ECFPs. G5 and P2 are the most similar compounds in the ligand set, and the similar fragments of them are marked in red.

Although TarPred and CPI-IGAE both utilize the ligand-based representations for proteins, TarPred uses the KNN algorithm for data mining while CPI-IGAE uses the GNN architecture. GNNs can deeply explore the overall association information among the data and learn meaningful task-specific node representations in an end-to-end manner. According to the embedding visualization shown in Figure 4C, targets within the same classes are spatially grouped, indicating that CPI-IGAE can learn distinguishable characteristics of different proteins.

#### Comparison with network-based methods

We then compared CPI-IGAE with some heterogeneous network-based methods, including DT-Hybrid [27], BLMNII [21], Heterogeneous Network Model (HNM) [39], Multiple Similarities Collaborative Matrix Factorization (MSCMF) [40], NetLapRLS [41], DTINet [22], NeoDTI [23] and End-to-End heterogeneous Graph representation learning-based framework for Drug-Target Interaction prediction (EEG-DTI) [20]. This comparison is conducted on the dataset from NeoDTI. The details of the experiment setting can be found in Supplementary Document Section 4.2 available online at http://bib.oxfordjournals.org/. Figure 2C and F shows that CPI-IGAE outperformed these baselines, with significant improvement (5% in terms of AUPRC and 3% in terms of AUROC) over the second-best method NeoDTI (the specific values are shown in Supplementary Table S3 available online at http://bib.oxfordjournals.org/). The results demonstrate the effectiveness of our specially designed homogeneous graph. We also conducted performance comparisons on several challenging scenarios provided by the original NeoDTI paper, which can be found in Supplementary Section 4.2 available online at http://bib.oxfordjournals.org/.

#### Comparison with molecular docking

We also conducted virtual screening on the collected LIT-PCBA dataset using CPI-IGAE and a molecular docking method Surflex-Dock (v.3066) [42]. The details of the experiment setting can be found in Supplementary Section 4.3 available online at http://bib.oxfordjournals.org/. The enrichment factors in true actives at a constant 1% false positive rate (EF1%) was chosen as the performance indicator. As Table 1 shows, the average of all the results of CPI-IGAE is close to that of the molecular docking. This comparison demonstrates that CPI-IGAE has competitive performance with molecular docking. Moreover, for some targets such as Estrogen receptor alpha (ESR1), Mechanistic target of rapamycin (MTORC1) and vitamin D3 receptor (VDR), CPI-IGAE performs better than molecular docking, while for some targets like Aldehyde dehydrogenase 1 (ALDH1), Glucocerebrosidase (GBA) and Pyruvate kinase muscle isoform 2 (PKM2), molecular docking performs better than CPI-IGAE. This indicates that our model can be complementary with traditional computational methods in CPI prediction task.

**Table 1:** Comparison results of CPI-IGAE and molecular docking

Target	Ligands in the train set	Actives: decoys	Max_sim to the train set (actives: decoys)	CPI-IGAE EF1%	Molecular docking (Surflex-Dock v.3066) EF1%
ADRB2	82	4: 78120	0.1633: 0.2110	0	0
ALDH1	7	1344: 25868	0.1829: 0.1745	0.744	1.25 ± 0.23
ESR1	240	28: 2632	0.2309: 0.2172	3.679	Agonist: 0.00/Antagonist: 1.60 ± 2.03
FEN1	5	92: 88850	0.1524: 0.1548	1.087	3.26
GBA	25	41: 74013	0.1725: 0.1711	0	4.47 ± 3.59
IDH1	3	9: 90512	0.1403: 0.1580	0	0.79 ± 2.97
MAPK1	89	77: 15657	0.2186: 0.2133	1.302	1.99 ± 1.38
MTORC1	210	24: 8243	0.2247: 0.2283	4.201	1.52 ± 2.10
OPRK1	288	6: 67454	0.3336: 0.2379	0	0
PKM2	5	136: 61380	0.1630: 0.1579	0	0.90 ± 0.61
PPARG	140	6: 1302	0.3408: 0.1953	16.77	5.56 ± 8.13
VDR	18	165: 66635	0.1378: 0.1525	3.636	0
Average	–	–	–	2.618	1.64 ± 1.62

IDH1, Isocitrate dehydrogenase; OPRK1, Kappa opioid receptor; PPARG, Peroxisome proliferator-activated receptor γ

### Model ablation study

To investigate which part of the specially designed homogeneous graph model attributes to its effectiveness, we further conducted an ablation study with the following setup:

(i) No EdgeWeight: we removed the edge weights to form an unweighted homogeneous graph.
(ii) No ProteinNode: we removed the protein nodes to construct a different homogeneous graph with only compound nodes, and the CPI prediction task was converted to a multilabel node classification task where the node labels are the targets of compounds.
(iii) Hinsage: we replaced the ligand-based ECFP-like vectors with sequence-based vectors for protein node features to construct a heterogeneous graph. The sequence-based vectors were generated from POSSUM [43] and were then cleaned to remove the highly correlated bits to form 420 bit vectors. HinSAGE is a variant of GraphSAGE extended for heterogeneous graphs, which was developed in StellarGraph (version 0.11.1) [44].

As shown in Figure 3D, these ablation procedures significantly compromised the performance of CPI-IGAE. From ‘-No EdgeWeight’, we can see the importance of edge weights, which can provide useful initial information to improve the accuracy and speed of training. The ‘-No ProteinNode’ and ‘-Hinsage’ demonstrate the protein features, especially our specially designed ligand-based protein features, are essential for the effectiveness of our homogeneous graph and can explain the performance improvements of CPI-IGAE over the other protein sequence-based methods and heterogeneous graph-based methods.

### Embedding visualization

We employed the UMAP to visualize the node embeddings learned by CPI-IGAE. As the regularized dot product used in our decoder can reflect the angular association between a target and ligand embedding, the vectors were projected onto a hypersphere by UMAP with the Haversine metric to measure their distances on a sphere [45]. Figure 4A shows the visualization of randomly selected 12 targets and their ligands, and we also transformed the 3D terrestrial globe to a 2D map and obtained the result as shown in Figure 4B. As expected, these results show that targets and their ligands are clustered together, which can demonstrate that CPI-IGAE can learn meaningful task-specific node representations. This is attributed to the ligand-based protein representations of our homogeneous graph.

We further visualized the protein embeddings in the same way. Figure 4C shows the embeddings of GPCRs and kinases, which reveals that targets within the same classes are spatially grouped. This is also the reason for the aforementioned robustness of CPI-IGAE to various protein families, i.e., CPI-IGAE can learn the characteristics of different proteins to distinguish them correctly. However, there are some nodes mixed up with the other class, and the hierarchical clustering tree of their embeddings is consistent with the visualization (Figure 4D). In contrast, the sequence-based phylogenetic tree generated from multiple sequence alignment(MSA) [46] separates GPCRs and kinases clearly (Figure 4E). As CPI-IGAE takes ligand-based representations for protein nodes to create a homogeneous graph, the protein embeddings of it can reflect the information of protein pockets to a certain extent, thereby making the embedding-based clustering differ greatly from the sequence-based clustering.

To prove this, the Euclidean distance between the 3D-structure-based pocket vectors of these proteins were calculated. Based on the 3D protein structures collected from the Protein Data Bank [47] and the AlphaFold protein structure database [48], the pocket-related parameters were calculated by SiteMap (Schrödinger Suite 2017) and then were normalized and concatenated to obtain the pocket vectors. As a heatmap shown in Figure 4F, the pocket vectors have smaller distances (deeper colors) for the proteins being clustered into the same group in Figure 4D. For example, Phosphatidylinositol 3-kinase catalytic subunit type 3 (PIK3C3) (kinase) and G-protein coupled receptor 55 (GPR55) (GPCR) have quite different sequences but are clustered together by their embeddings. Their pockets are both formed between the helices and are in similar shape and size (Figure 4G), which makes their ligands share similar structures. As shown in Figure 4H, some ligands of PIK3C3 and GPR55 are clustered closely through hierarchical clustering using ECFPs.

**Table 2:** Top 20 predict scores of the DrugBank dataset

DrugBank_ID	Protein_name (Gene_name)	Predict_score	Verification
**DB04617**	**Cholinesterase (BCHE)**	**1.0**	**Proved in the literature [** **49****] (the BCHE protein was from equine serum)**
DB04669	Mitogen-activated protein kinase 14 (MAPK14)	1.0	Unproved
DB06713	AR	1.0	Verified by DrugBank
**DB00294**	**AR**	**1.0**	**Proved in the literature [** **50****]**
DB00367	AR	1.0	Verified by DrugBank
DB11619	AR	1.0	Verified by DrugBank
DB07356	Dipeptidyl peptidase 4 (DPP4)	1.0	Verified by DrugBank
DB07356	Dipeptidyl peptidase 8 (DPP8)	1.0	Unproved
DB08208	Dual specificity mitogen-activated protein kinase kinase 1 (MAP2K1)	1.0	Verified by DrugBank
DB06813	Thymidylate synthase	1.0	Verified by DrugBank
DB06321	ALK tyrosine kinase receptor (ALK)	1.0	Unproved
DB07211	Cathepsin S (CTSS)	1.0	Unproved
**DB00091**	**CTSL**	**1.0**	**Proved in the literature [** **56****,** **57****] (molecular docking and MD simulation)**
DB08755	Cathepsin D	1.0	Unproved
DB08755	Cathepsin K	1.0	Unproved
DB08755	CTSS	1.0	Verified by DrugBank
DB08755	CTSL	1.0	Unproved
DB08755	Calpain-1 catalytic subunit	1.0	Unproved
DB08755	CTSB	1.0	Unproved
DB00910	VDR	1.0	Verified by DrugBank

*Note*: Bold entries indicate that the new CPI was verified in the literature. ALK, Anaplastic lymphoma kinase

Taken together, the visualization analysis demonstrates that CPI-IGAE can learn the protein embeddings that implicitly represent the features of the binding pocket as it creates a homogeneous graph by using ligand-based protein features. Therefore, CPI-IGAE can project the embedding of targets and their ligands onto closer points and thus outperform the methods based on protein sequences as shown in the ablation test.

### Some of the predicted novel CPIs were verified in the literature

We list the top 20 CPIs with highest predict scores in the DrugBank dataset (Table 2), and there are eight CPIs already existing in the database which are marked as ‘Verified by DrugBank’. Among the rest of the predicted novel CPIs, three pairs can be supported by previous studies in the literature:

(i) DB04617 is an experimental compound with a target of acetylcholinesterase in DrugBank. CPI-IGAE predicts DB0416 interacts with butyrylcholinesterase (BCHE), and this can be verified by a previous work [49], although the BCHE protein in this study was from equine serum.
(ii) DB00294 is an approved progesterone receptor agonist for long-acting contraception, which is also known as etonogestrel. CPI-IGAE predicts DB00294 interacts with an androgen receptor (AR), and this can be verified by a previous work [50].
(iii) The most worth mentioning is that CPI-IGAE predicts DB00091 interacts with Procathepsin L (CTSL). DB00091, also named cyclosporine, is an approved calcineurin inhibitor known for its immunomodulatory properties that prevent organ transplant rejection and treat various inflammatory and autoimmune conditions. Many studies [51–54] including clinical research studies have proved that cyclosporine is a therapeutic drug agonist for coronavirus disease 2019 (COVID-19), which is an ongoing global pandemic caused by severe acute respiratory syndrome coronavirus 2 (SARS-CoV-2). Meanwhile, CTSL plays an essential role in the entry of SARS-Cov-2 into the host [55]. This predicted novel CPI may provide an insight into the therapeutic of cyclosporine for COVID-19 and has been verified through the molecular docking and molecular dynamics (MD) simulations in the literature [56, 57].

Specially, the targets of the novel CPI such as AR [58], VDR [59], CTSL and Cathepsin B (CTSB) [60] have been reported to be relevant for the treatment of COVID-19, thus these new predicted CPIs that have not been verified are good candidates for wet experiment exploration. Overall, these novel CPIs predicted by CPI-IGAE with literature supports further prove the strong predictive power of this model.

## Conclusion

In this paper, we propose an IGAE-based model, named CPI-IGAE, for the CPI prediction task. To overcome the challenges in heterogeneous graph representation learning, a homogeneous graph was transformed from the compound–protein heterogeneous graph by integrating the ligand-based protein representation and overall similarity associations. The low-dimensional node embeddings are learned by IGAEs based on the homogeneous graph in an end-to-end manner. Moreover, it can be applied for new compounds outside the modeling dataset.

Via comprehensive performance comparisons, we show empirically that CPI-IGAE outperforms some state-of-the-art methods for CPI prediction. The ablation tests and analysis of embeddings obtained from the model further demonstrate the effectiveness of our method. Moreover, some of the predicted CPIs are verified in the literature, which indicates its ability to provide potential CPI candidates for further studies. Therefore, we believe that CPI-IGAE is a powerful and practical tool for CPI prediction, which can promote the development of drug discovery and drug repurposing. In the future, we will develop CPI-IGAE to incorporate more information in a better homogeneous way into the graph. For example, as the model shows the ability of characterizing the binding pockets to some extent, introducing pocket information to protein feature may further benefit the prediction of less-studied proteins.

**Key Points**

- To better conduct message passing and aggregating in graph, we transformed the heterogeneous graph to a homogeneous graph with directed and weighted edges.
- We adapted the inductive aggregators from GraphSAGE to fit the CPI prediction task and this enables our methods to predict CPIs outside the modeling dataset, which improves the generalization ability of this method.
- We proposed an end-to-end framework which can help learn the task-specific node embeddings for CPI prediction.
- The comprehensive performance evaluations of this model indicate that CPI-IGAE outperforms some state-of-the-art CPI prediction methods.

## Supplementary Material

SI_revised_bbac073

## Data Availability

Data and code can be found from this link https://github.com/wanxiaozhe/CPI-IGAE.

## Notes

**Xiaozhe Wan** is a PhD student at Shanghai Institute of Materia Medica. Her research interest is artificial intelligence-assisted drug design. Her affiliation is State Key Laboratory of Drug Research, Drug Discovery and Design Center, Shanghai Institute of Materia Medica, Chinese Academy of Sciences, 555 Zuchongzhi Road, Shanghai 201203, China; University of Chinese Academy of Sciences, No.19A Yuquan Road, Beijing 100049, China.

**Xiaolong Wu** is a PhD student at Shanghai Institute of Materia Medica. His research interests are artificial intelligence-assisted drug design and bioinformatics. His affiliation is State Key Laboratory of Drug Research, Drug Discovery and Design Center, Shanghai Institute of Materia Medica, Chinese Academy of Sciences, 555 Zuchongzhi Road, Shanghai 201203, China; School of Pharmacy, East China University of Science and Technology, Shanghai 200237, China.

**Dingyan Wang** is a PhD student at Shanghai Institute of Materia Medica. His research interest is artificial intelligence-assisted drug design. His affiliation is State Key Laboratory of Drug Research, Drug Discovery and Design Center, Shanghai Institute of Materia Medica, Chinese Academy of Sciences, 555 Zuchongzhi Road, Shanghai 201203, China; University of Chinese Academy of Sciences, No.19A Yuquan Road, Beijing 100049, China.

**Xiaoqin Tan** got her PhD degree at Shanghai Institute of Materia Medica in 2021. Her research interest is artificial intelligence-assisted drug design. Her affiliation is ByteDance AI Lab, Shanghai 201103, China.

**Xiaohong Liu** got his PhD degree at Shanghai Institute of Materia Medica in 2021. His research interest is artificial intelligence-assisted drug design. His affiliation is AlphaMa Inc., No. 108, Yuxin Road, Suzhou Industrial Park, Suzhou 215128, China.

**Zunyun Fu** is a PhD student at Shanghai Institute of Materia Medica. Her research interests are artificial intelligence-assisted drug design and quantum calculation. Her affiliation is State Key Laboratory of Drug Research, Drug Discovery and Design Center, Shanghai Institute of Materia Medica, Chinese Academy of Sciences, 555 Zuchongzhi Road, Shanghai 201203, China.

**Hualiang Jiang** is an academician medicinal scientist of the Chinese Academy of Sciences. His research interests are artificial intelligence-assisted drug design, computational chemistry, computational biology and pharmaceutical chemistry. His affiliation is State Key Laboratory of Drug Research, Drug Discovery and Design Center, Shanghai Institute of Materia Medica, Chinese Academy of Sciences, 555 Zuchongzhi Road, Shanghai 201203, China; University of Chinese Academy of Sciences, No. 19A Yuquan Road, Beijing 100049, China; School of Life Science and Technology, ShanghaiTech University, 393 Huaxiazhong Road, Shanghai 200031, China.

**Mingyue Zheng** is a professor at Shanghai Institute of Materia Medica. His research interests are artificial intelligence-assisted drug design, computational chemistry and computational biology. His affiliation is State Key Laboratory of Drug Research, Drug Discovery and Design Center, Shanghai Institute of Materia Medica, Chinese Academy of Sciences, 555 Zuchongzhi Road, Shanghai 201203, China; University of Chinese Academy of Sciences, No. 19A Yuquan Road, Beijing 100049, China.

**Xutong Li** is a postdoctoral researcher at Shanghai Institute of Materia Medica. Her research interest is artificial intelligence-assisted drug design. Her affiliation is State Key Laboratory of Drug Research, Drug Discovery and Design Center, Shanghai Institute of Materia Medica, Chinese Academy of Sciences, 555 Zuchongzhi Road, Shanghai 201203, China; University of Chinese Academy of Sciences, No. 19A Yuquan Road, Beijing 100049, China.
