# Supplementary material for: An inductive graph neural network model for compound–protein interaction prediction based on a homogeneous graph
Source: Brief Bioinform. 2022 Mar 12;23(3):bbac073. doi: 10.1093/bib/bbac073 (PMC9310259; doi:10.1093/bib/bbac073)
Supplement: SI_revised_bbac073 [file si_revised_bbac073.docx]

**Supplementary Information (SI Appendix)**

**An inductive graph neural network model for compound-protein interaction prediction based on a homogeneous graph**

Xiaozhe Wan, Xiaolong Wu, Dingyan Wang, Xiaoqin Tan, Xiaohong Liu, Zunyun Fu, Hualiang Jiang, Mingyue Zheng, Xutong Li.

**Corresponding authors:**

**Xutong Li**, Drug Discovery and Design Center, State Key Laboratory of Drug Research, Shanghai Institute of Materia Medica, Chinese Academy of Sciences, 555 Zuchongzhi Road, Shanghai 201203, China; University of Chinese Academy of Sciences, No.19A Yuquan Road, Beijing 100049, China. **E-mail**: [lixutong@simm.ac.cn](mailto:lixutong@simm.ac.cn)

**Mingyue Zheng**, Drug Discovery and Design Center, State Key Laboratory of Drug Research, Shanghai Institute of Materia Medica, Chinese Academy of Sciences, 555 Zuchongzhi Road, Shanghai 201203, China; University of Chinese Academy of Sciences, No.19A Yuquan Road, Beijing 100049, China. **E-mail**: [myzheng@simm.ac.cn](mailto:myzheng@simm.ac.cn)

**Xiaozhe Wan** is a Ph.D. student at Shanghai Institute of Materia Medica. Her research interest is artificial intelligence-assisted drug design. Her affiliation is Drug Discovery and Design Center, State Key Laboratory of Drug Research, Shanghai Institute of Materia Medica, Chinese Academy of Sciences, 555 Zuchongzhi Road, Shanghai 201203, China; University of Chinese Academy of Sciences, No.19A Yuquan Road, Beijing 100049, China.

**Xiaolong Wu** is a Ph.D. student at Shanghai Institute of Materia Medica. His research interests are artificial intelligence-assisted drug design and bioinformatics. His affiliation is Drug Discovery and Design Center, State Key Laboratory of Drug Research, Shanghai Institute of Materia Medica, Chinese Academy of Sciences, 555 Zuchongzhi Road, Shanghai 201203, China; School of Pharmacy, East China University of Science and Technology, Shanghai 200237, China.

**Dingyan Wang** is a Ph.D. student at Shanghai Institute of Materia Medica. His research interest is artificial intelligence-assisted drug design. His affiliation is Drug Discovery and Design Center, State Key Laboratory of Drug Research, Shanghai Institute of Materia Medica, Chinese Academy of Sciences, 555 Zuchongzhi Road, Shanghai 201203, China; University of Chinese Academy of Sciences, No.19A Yuquan Road, Beijing 100049, China.

**Xiaoqin Tan** got her Ph.D. degree at Shanghai Institute of Materia Medica in 2021. Her research interest is artificial intelligence-assisted drug design. Her affiliation is ByteDance AI Lab, Shanghai 201103, China.

**Xiaohong Liu** got his Ph.D. degree at Shanghai Institute of Materia Medica in 2021. His research interest is artificial intelligence-assisted drug design. His affiliation is AlphaMa Inc., No. 108, Yuxin Road, Suzhou Industrial Park, Suzhou 215128, China.

**Zunyun Fu** is a Ph.D. student at Shanghai Institute of Materia Medica. Her research interests are artificial intelligence-assisted drug design and quantum calculation. Her affiliation is Drug Discovery and Design Center, State Key Laboratory of Drug Research, Shanghai Institute of Materia Medica, Chinese Academy of Sciences, 555 Zuchongzhi Road, Shanghai 201203, China.

**Hualiang Jiang** is an academician medicinal scientist of the Chinese Academy of Sciences. His research interests are artificial intelligence-assisted drug design, computational chemistry, computational biology and pharmaceutical chemistry. His affiliation is Drug Discovery and Design Center, State Key Laboratory of Drug Research, Shanghai Institute of Materia Medica, Chinese Academy of Sciences, 555 Zuchongzhi Road, Shanghai 201203, China; University of Chinese Academy of Sciences, No.19A Yuquan Road, Beijing 100049, China; School of Life Science and Technology, ShanghaiTech University, 393 Huaxiazhong Road, Shanghai 200031, China.

**Mingyue Zheng** is a professor at Shanghai Institute of Materia Medica. His research interests are artificial intelligence-assisted drug design, computational chemistry and computational biology. His affiliation is Drug Discovery and Design Center, State Key Laboratory of Drug Research, Shanghai Institute of Materia Medica, Chinese Academy of Sciences, 555 Zuchongzhi Road, Shanghai 201203, China; University of Chinese Academy of Sciences, No.19A Yuquan Road, Beijing 100049, China.

**Xutong Li** is a postdoctoral researcher at Shanghai Institute of Materia Medica. Her research interest is artificial intelligence-assisted drug design. Her affiliation is Drug Discovery and Design Center, State Key Laboratory of Drug Research, Shanghai Institute of Materia Medica, Chinese Academy of Sciences, 555 Zuchongzhi Road, Shanghai 201203, China; University of Chinese Academy of Sciences, No.19A Yuquan Road, Beijing 100049, China.

In this supplementary document, we introduce six parts. In section 1, we introduce the details of the homogeneous graph construction. In section 2, we introduce the adjustments that our inductive graph aggregators made for GraphSAGE. In section 3, we introduce the optimization of our model. In section 4, we introduce the detail experiment settings of the model comparisons. In section 5, we simply describe the druggability of the protein targets in the modeling dataset.

**1. Construction of a homogeneous graph.**

**1.1 The effect of different contruction methods of protein node features.**

In the homogeneous graph, the 1024-bit vector for each protein node is constructed by its ligands in the training dataset. For each bit of this vector, if more than 1/3 of its ligands in the training dataset are 1 in this bit, it is set to 1, otherwise 0. We have tried the strategies of ‘1/4’, ‘3/4’, ‘1/2’, ‘2/3’, ‘max’ and ‘mean’, in which ‘max’ means that the bit of the constructed protein feature is set to 1 if one of its ligands in the training set is 1, and ‘mean’ means that the value of each bit in the constructed protein features is the average of the values at this bit of all its ligands in the training dataset. Then we have compared performance of models with these strategies on validation set. As shown in Figure S1, ‘1/3’ was chosen due to the best performance.


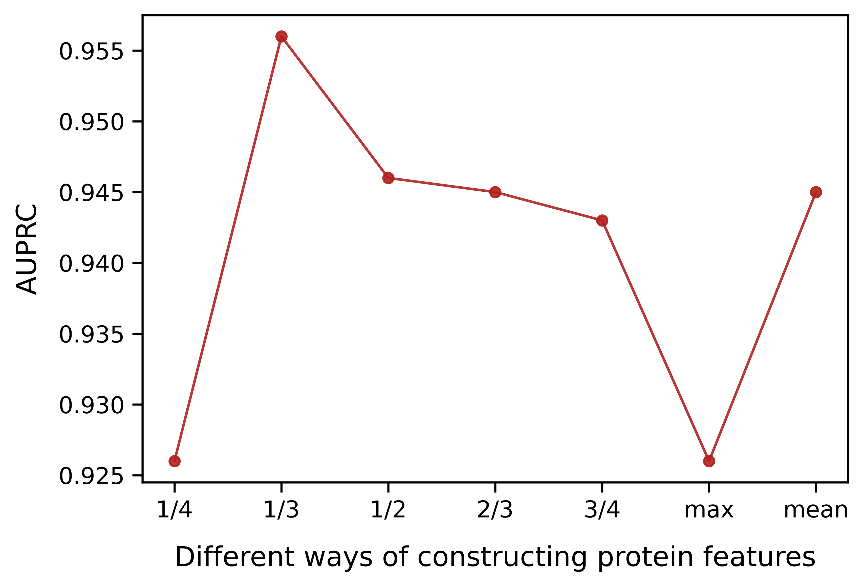


**Figure S1. Model performance on the validation set with respect to the different construction methods of protein node features.**

**1.2 The effect of edge numbers.**

In the homogeneous graph, for each node, 40 incoming edges to it were constructed from the top 20 compound nodes and the top 20 protein nodes with the largest DSCs to it. We also tried ‘top 5’, ‘top 10’, ‘top 15’, ‘top 25’ and ‘top 30’, and then compared the model performance and runtime on validation set. As shown in Figure S2, ‘top 20’ was chosen due to the best tradeoff between performance and runtime.


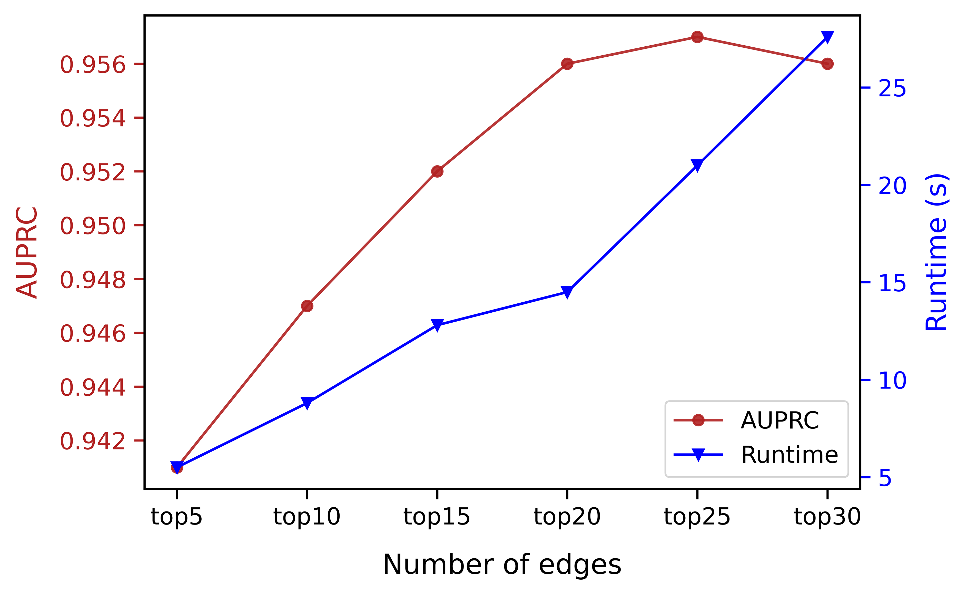


**Figure S2. Model performance on the validation set (red) and the runtime of each epoch (blue) with respect to the number of edges, respectively.**

**1.3 Generation of negative CPIs.**

One major problem for training a CPI prediction model is the lack of negative samples, which refers to the compound-protein pairs verified to be non-interactive by experiments. In this work, negative samples were generated by adjusted negative sampling which has been widely used in previous target prediction studies[1-3]. For each compound $m_{i}$, its constructed negative samples were generated according to the following steps:

(i) For each target $t_{j}$, calculate the DSC between the 1024-bit features of $m_{i}$ and $t_{j}$.

(ii) Select the $t_{j}$ where $DSC\left( m_{i},t_{j} \right)\leq0.4$ to form a candidate negative target set $T_{N}$.

(iii) Remove the positive targets of $m_{i}$ in $T_{N}$.

(iv) Randomly select $n_{N} (=f\times n_{p})$ targets in $T_{N}$, where $f$ is the ratio of negative samples to positive samples, and $n_{p}$ is the number of positive targets of $m_{i}$. We set $f=10$ to mimic the typical application scenario in which CPIs are sparsely labeled[3].

Step (ii) further improves the quality of these constructed negatives by limiting the DSCs to a small value, which ensure that the selected negatives are not similar to the ligands of the selected proteins and reduce including potential false positives.

**2.** **The adjustments of our inductive graph aggregators made for GraphSAGE.**

The inductive graph aggregator inspired by GraphSAGE enables the CPI-IGAE to predict new CPIs outside the modeling dataset. As shown in Figure S3, some adjustments were made for the GraphSAGE aggregators to better meet our requirements: (i) Firstly, the uniform neighbors sampling before aggregating in GraphSAGE is removed. Instead, the full set of neighbors is used to get stable and reproducible predictions. Specially, it should be pointed out that the neighbors of one node in this directed graph are all the source nodes of its incoming edges. The directed edges can prevent information redundancy caused by repeated aggregations. (ii) Moreover, the weights of these incoming edges are added to the aggregators in CPI-IGAE, which can provide useful initial information to improve the accuracy and speed of training.


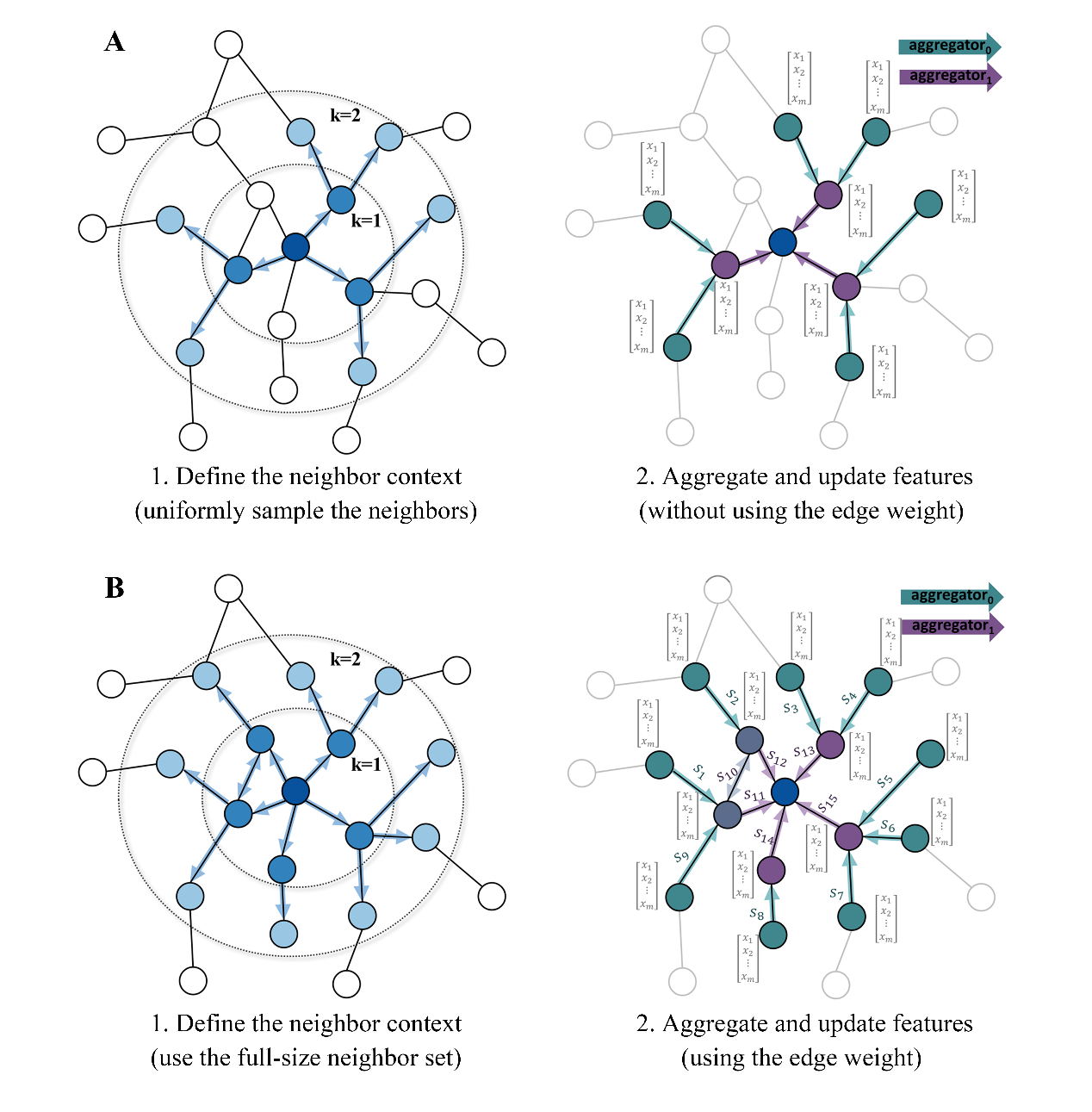


**Figure S3. Comparison of the graph aggregators in GraphSAGE and CPI-IGAE.**

(A) Two steps of the GraphSAGE aggregators: (i) uniform sampling a fix-sized neigbors in each depth (in this example, size = 3 when k = 1, size = 2 when k = 2) to define the context; (ii) aggregating the information of neighbors in the context and updating the embedding of the target node.

(B) Two steps of CPI-IGAE aggregators: (i) instead of sampling the neighbors, CPI-IGAE uses the full-size neighbors; (ii) CPI-IGAE uses the edge weights when aggregating the neighbor information.

**3. Model optimization.**

The model was trained on all positive pairs and the constructed negative pairs in the training set, using the Adam optimizer for back-propagation to tune the parameter matrices. Early stopping was used to alleviate overfitting if no further improvement in the performance of the model was shown on the validation set in 20 successive steps. The performance was evaluated by AUPRC, which provides a more informative criterion than AUROC on this imbalance datasets[4]. To optimize the generalization ability of our model, we performed a hyperparameter sweep on the combinations of all the hyperparameters listed in Table S1 through a grid search. The AUPRC of validation set was used to choose the best group of these hyperparameters as in the early stopping.

CPI-IGAE model is developed in DGL[5] (version 0.4.3) and PyTorch[6] (version 1.4.0). The computational performance took about 2 hours to train the model (through 450 epochs and 15 seconds each) with a NVIDIA TITAN RTX GPU on an Intel platform.

**Table S1. Hyperparameters of CPI-IGAE.**

| Hyperparameters | Choices | Best |
| --- | --- | --- |
| Learning rate | 1e-3, 1e-4, 1e-5 | 1e-4 |
| Dropout | 0, 0.1, 0.2, 0.3, 0.4, 0.5 | 0.2 |
| Layer size | [256], [256,256], [256,256,256], [512], [512,512], [512,512,512], [1024], [1024,1024], [1024,1024,1024], [1024,512], [1000,500], [500,500] | [1000,500] |
| Batch size | 1024, 512, 256 | 512 |
| Embedding norm | True, False | False |
| ($\boldsymbol{\alpha, \gamma}$) | (0.25, 1.0), (0.25, 2.0), (0.5, 1.0),  (0.5, 2.0), (0.75, 1.0), (0.75, 2.0) | (0.25, 2.0) |

**4. Model comparisons.**

**4.1 Comparison with feature-based methods.**

For comparison with feature-based methods, the new compounds and their corresponding CPIs from DrugBank (v5.1.1)[7] and the TTD (v6.1.01)[8] were collected. The compounds overlapped with the modeling ChEMBL dataset were removed. In detail, the DrugBank dataset contains 2504 compounds and 4212 CPIs, and the TTD dataset contains 9322 compounds and 13063 CPIs. Figure S5A shows the distribution of the maximum Tanimoto similarities generated by comparing each compound in the external datasets with the training compounds, indicating that these two external datasets have different distributions from the modeling dataset and can provide convincing validations. The number of the negatives (generated by the aforementioned negative sampling) is 10 times of the positives. Eight models were chosen as baselines.

Random forest (RF) is a machine learning model and was implemented using scikit-learn[9]. Four hyperparameters were considered including ‘max_features’ , ‘max_depth’, ‘criterion’ and ‘n_estimators’. The best group of these hyperparameters is set ‘max_features’ to ‘log2’, ‘max_depth’ to 50, ‘criterion’ to ‘gini’ and ‘n_estimators’ to ‘100’.

Support vector machine (SVM) is implemented by the LinearSVC module of scikit-learn, which is a faster implemention of SVM for classification with a linear kernal. Two hyperparameters were considered: the penalty parameter C and the penalty type. The best group of these hyperparameters is set C to 1.0 and penalty type to ‘l1’.

Fully-connected neural network (FNN) is a classical deep learning model and was implemented using PyTorch. The considered hyperparameters include hidden layer size, dropout probability, learning rate and batch size, and the best group of them is set hidden layer size to [1000, 500], dropout probability to 0.2, learning rate to 1e-4, and batch size to 512.

SEA[10, 11] relates proteins based on the statistically calculated similarity of their respective ligands, and this method was further applied for target identification successfully. The equations for calculating the statistical scores can be found in the original works.

TarPred[12, 13] integrates K-Nearest Neighbors (KNN) algorithm with molecular similarity-based searching strategy for target identification. As demonstrated in the original work, K is set to 3.

DeepDTA[14] utilizes convolutional neural networks (CNNs) to extract low-dimensional embeddings of compounds and proteins, and then concatenated two embeddings of the compound-protein pair to feed into fully connected layers to calculate the final output. The hyperparameters were set as same as its original work.

GraphDTA[15] encodes a drug as an undirected graph with a feature map and an adjacent matrix. Graph neural networks is used to extract feature from drug graphs, and convolutional blocks is used to extract feature from protein sequences. The features of drugs and proteins are also combined to pass through fully connected layers to get the final outputs. The hyperparameters were set as same as its original work.

TransformerCPI[16] uses CNN and graph convolution network (GCN) to generate protein sequence representations and atom sequence representations, respectively. Then a transformer decoder is used to get the interaction features from these two kinds of sequences, where the protein sequences are used as original texts and the drug sequences are used as previous translations. The interaction features are subsequently fed into linear layers to output the interaction probabilities. The hyperparameters were set as same as its original work.

To conduct a fair comparison, each model was trained on the same train set of CPI-IGAE, and the hyperparameters were determined on the same validation set. Every model, except SEA and TarPred which have no trainable parameters, was performed three independent runs with different random seeds for training using the best set of hyperparameters.

**Table S2. Comparison results with feature-based methods.**

| Method | DrugBank dataset | | TTD dataset | |
| --- | --- | --- | --- | --- |
|  | AUPRC | AUROC | AUPRC | AUROC |
| RF | 0.336 ± 0.003 | 0.514 ± 0.002 | 0.324 ± 0.004 | 0.510 ± 0.001 |
| SVM | 0.545 ± 0.006 | 0.504 ± 0.006 | 0.545 ± 0.005 | 0.507 ± 0.004 |
| FNN | 0.257 ± 0.007 | 0.746 ± 0.005 | 0.276 ± 0.006 | 0.780 ± 0.004 |
| SEA | 0.657 | 0.708 | 0.716 | 0.745 |
| TarPred | 0.760 | 0.739 | 0.806 | 0.789 |
| DeepDTA | 0.606 ± 0.006 | 0.874 ± 0.004 | 0.702 ± 0.007 | 0.910 ± 0.004 |
| GraphDTA | 0.731 ± 0.006 | 0.914 ± 0.004 | 0.786 ± 0.007 | 0.937 ± 0.005 |
| TransformerCPI | 0.761 ± 0.007 | 0.920 ± 0.003 | 0.825 ± 0.005 | 0.940 ± 0.006 |
| **CPI-IGAE** | **0.813 ± 0.005** | **0.927 ± 0.003** | **0.875 ± 0.005** | **0.947 ± 0.002** |

**4.2 Comparison with heterogeneous network-based methods.**

For comparison with heterogeneous network-based methods, the dataset constructed in the study of NeoDTI[3] was used to follow the implement details of these baselines and thus draw parallel comparsions with them. In detail, the NeoDTI dataset contains 708 compounds, 1512 targets and 1923 CPIs. The number of the negative drug-protein pairs (generated by the aforementioned negative) is 10 times of the positive pairs. Eight previous reported methods were chosen as baselines:

DT-Hybrid[17] extends network-based inference (NBI) and the Hybrid algorithms by integrating previous domain-dependent knowledge through similarity matrix among drugs and targets.

BLMNII[18] combines the bipartite local model (BLM) with the neighbor-based interaction profile inferring (NII).

HNM[19] utilizes a random walk with restart (RWR) to iteratively propagate association information in the three-layer heterogeneous network consisting of three omics data about diseases, drugs and targets for drug repurposing.

MSCMF[20] employs matrix factorization to project drugs and targets of a given drug-target interaction (DTI) network into a common low-rank feature space, which is further consistent with the corresponding drug and protein similarity matrices obtained by integrating multiple data sources through a weighted averaging scheme.

NetLapRLS[21] applies Laplacian regularized least square (RLS) and incorporates both similarity and interaction kernels with bipartite graph as input for drug repurposing.

DTINet[22] first employs an unsupervised manner which combines RWR with a dimensional reduction scheme (DCA) to learn low-dimensional representations of drugs and targets from heterogeneous networks (e.g., drugs, diseases, targets and side-effects), and then finds an optimal projection from drug space into target space to enable the DTI prediction.

NeoDTI conducts an end-to-end GCNs to automatically learn the topoloty-preserving representation of each node to predict novel DTIs in the same heterogeneous network of DTINet.

EEG-DTI[23] utilizes an end-to-end learning framework based on heterogeneous graph convolution networks (HGCNs) to learn task-related node representations for DTI prediction.

Ten-fold cross-validation was performed, where in each fold a randomly chosen subset of 90% positive and negative links was used to train the models, and then the trained models were used to predict the existence of the remaining 10% links. The details on how to integrate the data and how to determine the hyperparameters can be found in the work of NeoDTI[3] and EEG-DTI[23]. For CPI-IGAE, the best set of hyperparameters described above was used.

**Table S3. Comparison results with network-based methods.**

| Method | NeoDTI dataset | |
| --- | --- | --- |
|  | AUPRC | AUROC |
| DT-Hybrid | 0.514 ± 0.007 | 0.833 ± 0.004 |
| BLMNII | 0.468 ± 0.005 | 0.853 ± 0.002 |
| HNM | 0.570 ± 0.007 | 0.888 ± 0.001 |
| MSCMF | 0.590 ± 0.052 | 0.832 ± 0.029 |
| NetLapRLS | 0.743 ± 0.014 | 0.903 ± 0.008 |
| DTINet | 0.811 ± 0.006 | 0.913 ± 0.002 |
| NeoDTI | 0.871 ± 0.004 | 0.957 ± 0.001 |
| EEG-DTI | 0.868 ± 0.006 | 0.956 ± 0.001 |
| **CPI-IGAE** | **0.924 ± 0.006** | **0.982 ± 0.002** |

Moreover, we also conducted the performance comparisons on several challenging scenarios provided by the original NeoDTI paper. These scenarios include: (i) treating all unknown drug-protein pairs as negative samples, (ii) removing DTIs with similar drugs (i.e. drug chemical structure similarities > 0.6) or proteins (i.e. protein sequence similarities > 40%), (iii) training on non-unique DTIs and then evaluating on unique DTIs, and (iv) removing DTIs with drugs sharing similar drug interactions (i.e. Jaccard similarities >0.6). However, the other two scenarios mentioned in the NeoDTI paper which are removing DTIs with drugs sharing similar side-effects interactions and removing DTIs with drugs or proteins sharing similar diseases are not conducted as our CPI-IGAE model do not contain the side-effect and disease entities. The experiment details are the same as described in the original NeoDTI paper. The results are shown in Figure S4.

As shown in Figure S4A and S4E, for scenario (i) of treating all unknown drug-protein pairs as negative samples, CPI-IGAE shows comparable performance with the best NeoDTI and outperforms the other models in terms of AUPRC, and AUROC is on average. As generating reliable negative data is more reasonable to simulate the real application scenario, rather than treating all unknown pairs as negative samples, we suppose that the moderate performance of CPI-IGAE on this scenario is acceptable. For scenario (ii) of removing DTIs with similar drugs or proteins (Figure S4B and S4F) and scenario (iii) of training on non-unique DTIs and then evaluating on unique DTIs (Figure S4C and S4G), our CPI-IGAE outperforms better than the other network-based methods. For scenario (iv) of removing DTIs with drugs sharing similar drug interactions (Figure S4D and S4H), although NeoDTI contains more information about drug-drug interaction and side-effects, the performance of CPI-IGAE is still slightly better than the second-best NeoDTI by only utilizing the drug-drug similarity information.


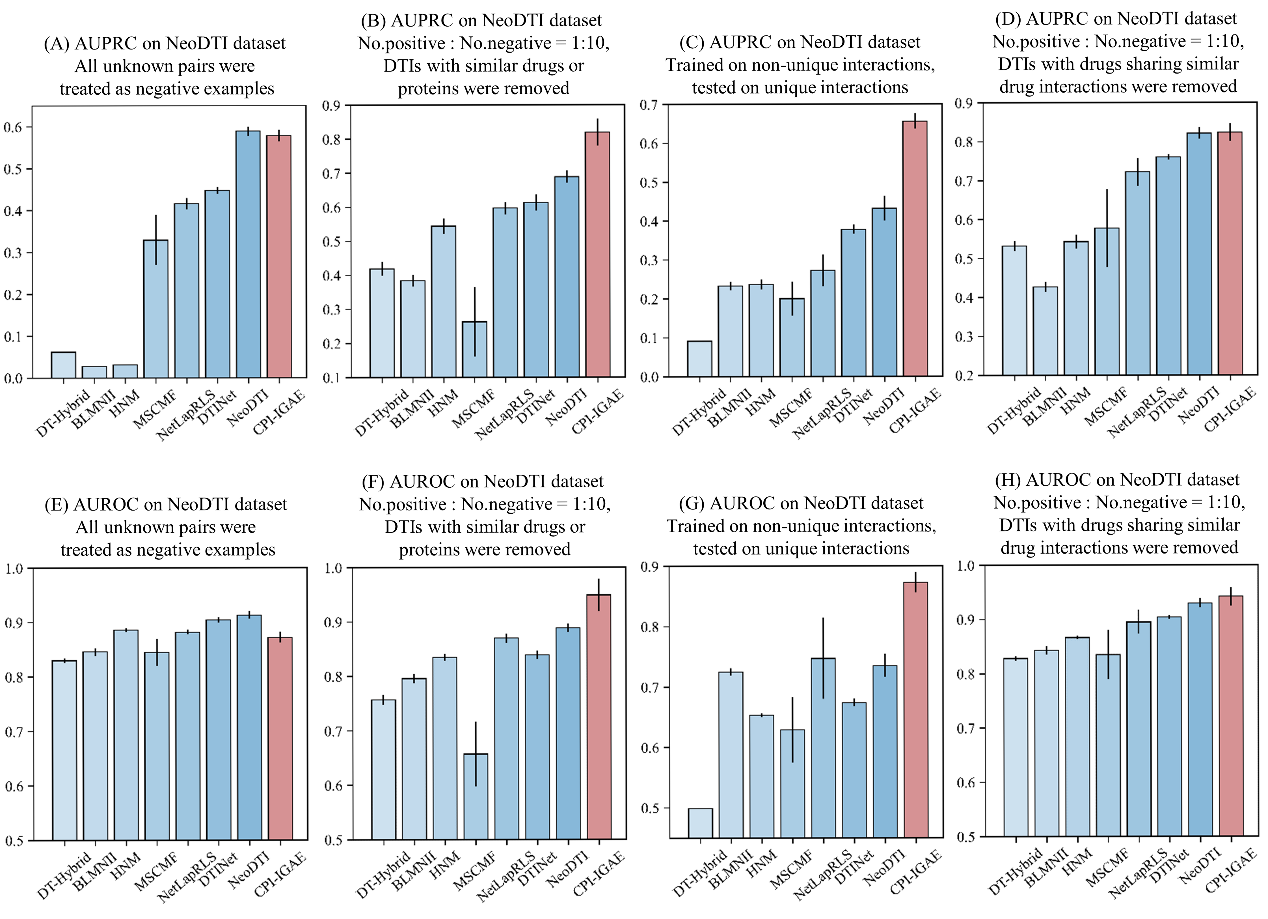


**Figure S4. Performance evaluation of CPI-IGAE with other network-based methods on several challenging scenarios in terms of AUPRC and AUROC scores.**

(A) AUPRCs and (E) AUROCs of a 10-fold cross-validation test in which all unknown drug–target interacting pairs were treated as negative samples.

(B) AUPRCs and (F) AUROCs of a 10-fold cross-validation test with positive: negative ratios = 1: 10 in which DTIs with similar drugs and proteins were removed.

(C) AUPRCs and (G) AUROCs of a test in which models were trained on non-unique drug–target interacting pairs and tested on unique drug–target interacting pairs.

(D) AUPRCs and (H) AUROCs of a 10-fold cross validation with positive: negative ratios = 1: 10 in which DTIs with drugs sharing similar drug interactions were removed.

**4.3 Comparison with molecular docking.**

Tran-Nguyen et al.[24] constructed a unbiased dataset LIT-PCBA based on the debiasing strategy asymmetric validation embedding (AVE) as a benchmark of machine learning and virtual screening. LIT-PCBA contains 15 targets with their active ligands and inactive decoys. The compounds of each target have been split into the training set and validation set with a ratio of ~3:1. The validation set was employed to conduct the performance comparison of CPI-IGAE and molecular docking. As histone acetyltransferase KAT2A (KAT2A) and cellular tumor antigen p53 (TP53) are not presented in our training set, we ignored these two targets in this comparison. Besides, LIT-PCBA divided estrogen receptor α (ESR1) into two groups: ESR with agonists (ESR_ago) and ESR with antagonists (ESR_antago). Since CPI-IGAE do not take the types of CPIs into account, the two targets ESR-ago and ESR_antago were merged together for calculation of CPI-IGAE. In detail, this dataset contains 12 targets along with their active ligands and inactive decoys. More details can be found in Table 1. Figure S5C shows the distributions of the maximum Tanimoto similarities of our training compounds to LIT-PCBA’s active ligands and inactive decoys, respectively. The maximum similarities are mainly in the range of 0.2 to 0.4, indicating that LIT-PCBA’s compounds are not similar to those in the training set and it is a convincing external test set. Moreover, the distribution of positive compounds and negative compounds in the PCBA dataset is close, which indicates that the results of virtual screening are not artifically inflated by the structural differences among compounds.

Moreover, we also conducted the ‘-No ProteinNode’ ablation test for the virtual screening on LIT-PCBA dataset because the the protein information for all CPIs in the virtual screening towards a certain target is all the same. This ablation test is the same as described in section 3.2 ‘Model ablation study’ of the main article. The results are shown in Table S4, and the decreased performance of CPI-IGAE without protein nodes demonstrates that the protein information is helpful for virtual screening.


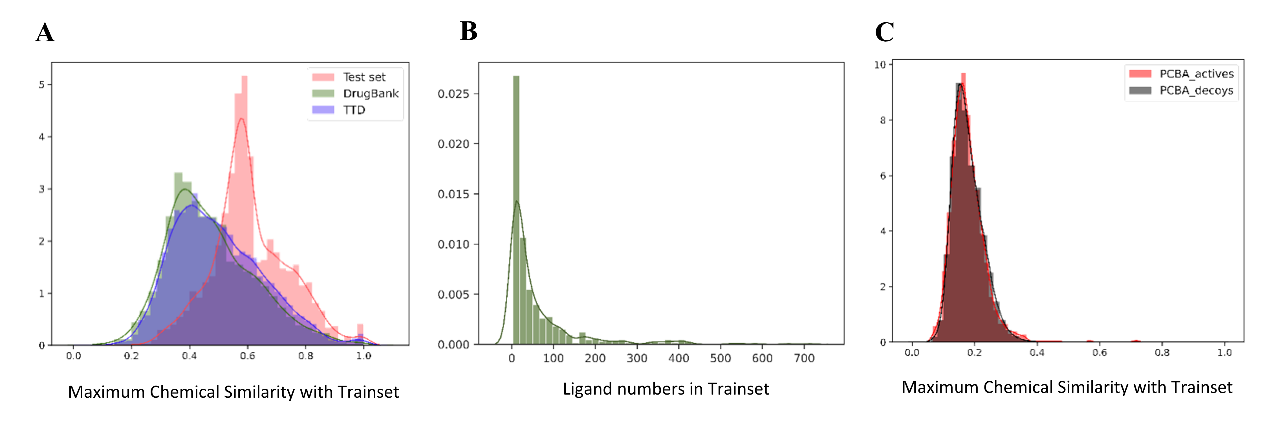


**Figure S5. Analysis of datasets for model comparison.**

(A) The distributions of the maximum Tanimoto similarities between each compound in the test set (red), DrugBank dataset (green) and TTD dataset (blue) with the training compounds.

(B) The distribution of the ligand numbers of targets in the train set.

(C) The distributions of the maximum Tanimoto similarities between each compound in the active ligands (red) and inactive decoys (black) of the 12 targets in the LIT-PCBA dataset with the training compounds.

**Table S4. Comparison results of CPI-IGAE and CPI-IGAE without protein node on LIT-PCBA dataset.**

| **Target** | **# Ligands in**  **train set** | **CPI-IGAE**  **EF_1%** | **CPI-IGAE**  **-No ProteinNode**  **EF_1%** |
| --- | --- | --- | --- |
| ADRB2 | 82 | 0 | 0 |
| ALDH1 | 7 | 0.744 | 0.596 |
| ESR1 | 240 | 3.679 | 0 |
| FEN1 | 5 | 1.087 | 1.087 |
| GBA | 25 | 0 | 0 |
| IDH1 | 3 | 0 | 0 |
| MAPK1 | 89 | 1.302 | 1.302 |
| MTORC1 | 210 | 4.201 | 0 |
| OPRK1 | 288 | 0 | 0 |
| PKM2 | 5 | 0 | 0.735 |
| PPARG | 140 | 16.77 | 0 |
| VDR | 18 | 3.636 | 1.818 |
| **Average** | **-** | **2.618** | **0.462** |

**5. Druggability of targets in the modeling dataset.**

We check the druggability of the 784 protein targets in the modeling dataset by calculating the intersections of them and the human targets contained in two public databases TTD (v8.1.01)[25] and Target Central Resource Database (TCRD, v6.12.4)[26], respectively. According to TTD, of the 784 targets, 280 targets are “successful targets” (targeted by at least one approved drug) and 334 targets have ligands in clinical trials, preclinical research or patents (Figure S6A). And according to TCRD, 304 of the 784 targets are the “Tclin” proteins and 477 are the “Tclin” proteins (Figure S6B), where “Tclin” are proteins which have approved drugs with known mechanism of action, and “Tchem” are proteins which have ligands with high potency. This analysis demonstrates that these 784 proteins in our modeling dataset have strong druggability and are worth for research.


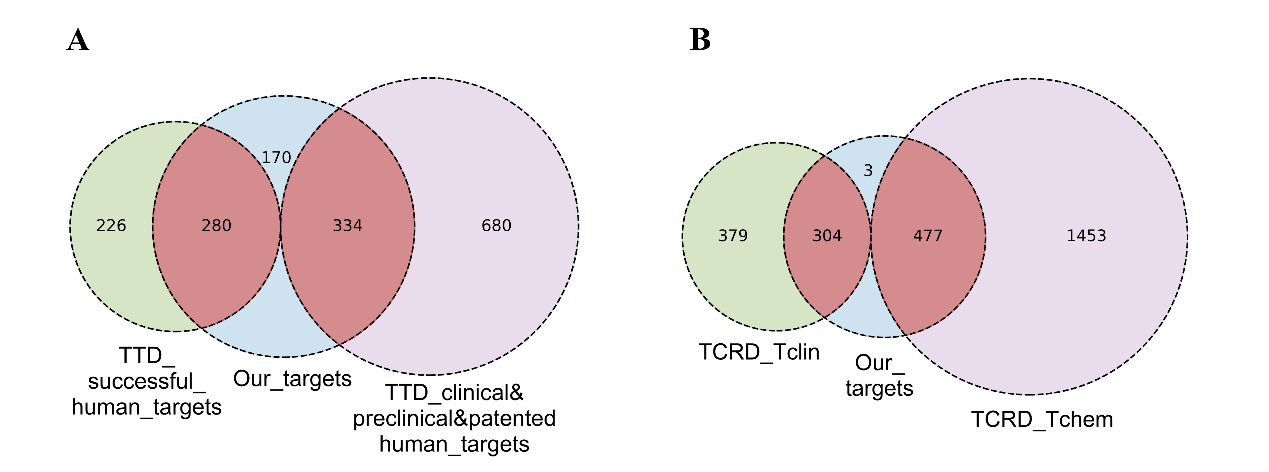


**Figure S6. Intersections of the 784 targets in the modeling dataset of CPI-IGAE and the targets contained in the databases including (A) TTD and (B) TCRD, respectively.**

**References**

1. Liu H, Sun J, Guan J et al. Improving compound–protein interaction prediction by building up highly credible negative samples, Bioinformatics 2015;31:i221-i229.

2. Jacob L, Vert J-P. Protein-ligand interaction prediction: an improved chemogenomics approach, Bioinformatics 2008;24:2149-2156.

3. Wan F, Hong L, Xiao A et al. NeoDTI: neural integration of neighbor information from a heterogeneous network for discovering new drug-target interactions, Bioinformatics 2019;35:104-111.

4. Saito T, Rehmsmeier M. The precision-recall plot is more informative than the ROC plot when evaluating binary classifiers on imbalanced datasets, PLOS ONE 2015;10:e0118432.

5. Wang M, Yu L, Zheng D et al. Deep Graph Library: Towards Efficient and Scalable Deep Learning on Graphs 2019.

6. Paszke A, Gross S, Massa F et al. Pytorch: An imperative style, high-performance deep learning library, Advances in neural information processing systems 2019;32:8026-8037.

7. Wishart DS, Feunang YD, Guo AC et al. DrugBank 5.0: a major update to the DrugBank database for 2018, Nucleic Acids Research 2017;46:D1074-D1082.

8. Li YH, Yu CY, Li XX et al. Therapeutic target database update 2018: enriched resource for facilitating bench-to-clinic research of targeted therapeutics, Nucleic Acids Research 2017;46:D1121-D1127.

9. Pedregosa F, Varoquaux G, Gramfort A et al. Scikit-learn: Machine learning in Python 2011;12:2825-2830.

10. Keiser MJ, Roth BL, Armbruster BN et al. Relating protein pharmacology by ligand chemistry, Nature Biotechnology 2007;25:197-206.

11. Keiser MJ, Setola V, Irwin JJ et al. Predicting new molecular targets for known drugs, Nature 2009;462:175-181.

12. Liu X, Gao Y, Peng J et al. TarPred: a web application for predicting therapeutic and side effect targets of chemical compounds, Bioinformatics 2015;31:2049-2051.

13. Liu X, Xu Y, Li S et al. In Silicotarget fishing: addressing a “Big Data” problem by ligand-based similarity rankings with data fusion, Journal of Cheminformatics 2014;6:33.

14. Öztürk H, Özgür A, Ozkirimli E. DeepDTA: deep drug–target binding affinity prediction, Bioinformatics 2018;34:i821-i829.

15. Nguyen T, Le H, Quinn TP et al. GraphDTA: predicting drug–target binding affinity with graph neural networks, Bioinformatics 2020;37:1140-1147.

16. Chen L, Tan X, Wang D et al. TransformerCPI: improving compound-protein interaction prediction by sequence-based deep learning with self-attention mechanism and label reversal experiments, Bioinformatics 2020;36:4406-4414.

17. Alaimo S, Pulvirenti A, Giugno R et al. Drug–target interaction prediction through domain-tuned network-based inference, Bioinformatics 2013;29:2004-2008.

18. Mei J-P, Kwoh C-K, Yang P et al. Drug–target interaction prediction by learning from local information and neighbors, Bioinformatics 2012;29:238-245.

19. Wang W, Yang S, Zhang X et al. Drug repositioning by integrating target information through a heterogeneous network model, Bioinformatics 2014;30:2923-2930.

20. Zheng X, Ding H, Mamitsuka H et al. Collaborative matrix factorization with multiple similarities for predicting drug-target interactions. In: Proceedings of the 19th ACM SIGKDD international conference on Knowledge discovery and data mining. 2013, p. 1025-1033.

21. Xia Z, Wu L-Y, Zhou X et al. Semi-supervised drug-protein interaction prediction from heterogeneous biological spaces, BMC Syst Biol 2010;4:S6.

22. Luo Y, Zhao X, Zhou J et al. A network integration approach for drug-target interaction prediction and computational drug repositioning from heterogeneous information, Nature Communications 2017;8:573.

23. Peng J, Wang Y, Guan J et al. An end-to-end heterogeneous graph representation learning-based framework for drug–target interaction prediction, Briefings in Bioinformatics 2021;22.

24. Tran-Nguyen V-K, Jacquemard C, Rognan D. LIT-PCBA: An unbiased data set for machine learning and virtual screening, J Chem Inf Model 2020;60:4263-4273.

25. Zhou Y, Zhang Y, Lian X et al. Therapeutic target database update 2022: facilitating drug discovery with enriched comparative data of targeted agents, Nucleic Acids Research 2021.

26. Sheils TK, Mathias SL, Kelleher KJ et al. TCRD and Pharos 2021: mining the human proteome for disease biology, Nucleic Acids Research 2020;49:D1334-D1346.
